# Preventing domestic accidents in families with children: a scoping review

**DOI:** 10.1515/med-2026-1446

**Published:** 2026-06-03

**Authors:** Sónia Cristina Fernandes da Silva, Laura Viegas, Fátima Rodrigues

**Affiliations:** Unidade de Cuidados de Saúde Personalizados Alhandra, Unidade Local de Saúde Estuário do Tejo, Lisboa, Portugal; Escola Superior de Enfermagem de Lisboa, Lisboa, Portugal

**Keywords:** family, accidents preventions, nurse, home environment

## Abstract

**Introduction:**

Nurses play a role in preventing domestic accidents involving children. We conducted a scoping review on the nursing interventions to prevent domestic accidents in families with children in the first year of life.

**Content:**

Scoping review based on the principles recommended by Joanna Briggs Institute, using CINAHL Complete e MEDLINE Complete data bases, through EBSCOhost platform (2018–2024), in natural and indexed language. Complemented research through Google Scholar.

**Summary and Outlook:**

Four articles met the study’s inclusion criteria. The articles analysed highlighted the importance of nurses as members of the healthcare team in collaboration with families in preventing domestic accidents. Nursing interventions include providing information about the types of domestic accidents, the risk factors and the child’s motor and cognitive development, as well as measures to prevent domestic accidents. The teaching methodology should combine an educational approach with an active and demonstrative methodology. Nurses have a leading role in preventing domestic accidents by empowering families to create a safe home environment. Through systematic interventions it’s possible to strengthen both parental competence and previously transmitted interventions.

## Introduction

Domestic accidents account for a high number of deaths worldwide [1], as well as years of life lost due to disabilities resulting from these accidents [2]. According to the Association for the Promotion of Child Safety “In Portugal, between 1992 and 2020, more than 6,500 children and young people died as a result of trauma and unintentional injury or accident.” [2] meaning “the loss of almost 380,000 potential years of life lost (INE) years in which children were unable to grow, learn and contribute to their community and society in general” [2]. In addition to deaths, there are thousands of hospitalizations annually due to domestic accidents, which, in many cases, leave irreversible consequences [3].

According to Trauma and Accident Epidemiology and Surveillance System – EVITA – “home” is the place where most accidents occur [4]. As “home” is a risky place, parental behaviour is a decisive factor in preventing domestic accidents, considering that it is the place where they spend most of their time with their children. The domestic family environment, architecturally created to meet the needs of adults, is not prepared for a child, and parents must ensure that all safety conditions are guaranteed. The responsibility of the parental role implies increased attention to children in the first year of life, because it is a stage of motor and cognitive development prone to discovery of the surrounding environment [5], 6], which if not adapted will, in itself, increase the risk of accidents [5], 6].

The domestic accidents that can occur at home are falls, burns, poisonings, drownings, asphyxia, suffocation and strangulation. In Portugal, the leading cause of death in the first year of life is asphyxia, suffocation and strangulation, with 13 out of 18 children dying from this cause [2]. Falls are the second leading cause of death from accidental or unintentional injury worldwide, followed by road accidents [7]. In Portugal, when analysing the number of calls to 112, forwarded by the National Institute of Medical Emergency – Urgent Patient Guidance Centre–between 2013 and 2021, it was found that 73 % of calls were due to falls, 10.4 % of which refer to the age group 0–4 years old [2]. In children, burns are the fifth most common cause of unintentional injuries occurring at home and can be caused by heat, radiation, electricity, friction or contact with chemicals [8]. There is an association between inadequate parental supervision and the increasing of these injuries [8]. They are by far the most distressing and painful, leading to prolonged hospitalizations and often resulting in long-term disfigurement and disability [8]. In Portugal, poisonings in children under 12 months of age do not represent the highest number of all poisonings recorded, however, in 2021, 538 children under 12 months of age were victims of poisoning [9]. In 2019, drowning accounted for 5 % of all deaths worldwide [1]. In Portugal, drowning accounts for 0.1 % of deaths in all age groups up to 18 years of age [2].

Despite the significant reduction in child and youth mortality due to trauma and unintentional injuries in Portugal over the last 30 years [2], there is still a long way to go in preventing them. Nurses, in their privileged position with families, should intervene in different contexts, adopting a proactive attitude, identifying risk factors, proposing changes and encouraging the adoption of safe behaviours.

Home visits, as a context for promoting and preventing disease, are an excellent way to bring nurses and families closer together [10], allowing to understand their reality in their own environment. We can say that nursing interventions with families with children in their first year of life will always have an educational, guiding and inclusive role, allowing families to make the decision to change the home environment.

## Objectives

To map in the scientific literature the nursing interventions to prevent domestic accidents in families with children in the first year of life.

## Methods

The scoping review was carried out based on the principles recommended by the Joanna Briggs Institute for scoping reviews [11]. Considering the mnemonic PCC (population, concept and context), the question that guided our research was “What are the nursing interventions for the prevention of domestic accidents in families with children in the first year of life?”. The following constituent elements of the PCC were identified: Population (P) – parents, family; Concept (C) – Nurse, nursing interventions, safety, safety accidents, injuries, injuries and wounds, unintentional injuries, accidents prevention and prevention & control; and Context (C) – home, home environment, accidents, home (Table 1).

**Table 1:** Strategy research.

	Natural language	CINAHL Complete	MEDLINE complete
Population	Parents	Parents	Parents
	Family	Family	Family
Concept	Nurse interventions	Nurse	Nurse
	Safety	Injuries	Injuries
	Safety accidents	Injuries and wounds	Injuries and wounds
		Unintentional injuries	Unintentional injuries
		Accidents prevention	Accidents prevention
		Prevention & control	Prevention & control
Context	Home	Home environment	Home environment
		Accidents, home	Accidents, home

### Inclusion criteria

Studies that identify nursing interventions in the prevention of domestic accidents in families with children under 12 months of age.

The inclusion criteria are literature reviews, studies in the quantitative, qualitative or mixed paradigm (both), primary or secondary, published between 2018 and 2024, in Portuguese, English and/or Spanish and with free full text availability.

### Types of participants (population)

Families with children under 12 months.

### Concept

Studies that include nursing interventions to prevent domestic accidents in families with children under 12 months old.

### Context

Studies that include nursing intervention in the prevention of accidents in the domestic environment.

### Types of sources of evidence

Includes all literature: literature reviews, studies in the quantitative, qualitative or mixed paradigm (both), primary or secondary.

### Search strategies

Three stages of search strategy are necessary for all types of reviews [11]. According to the study in question, the first stage involved an initial search limited to two databases, CINAHL and MEDLINE (using the EBSCOhost platform). The choice of two databases is appropriate for scoping reviews of tools that assess quality of life [11].

The initial research started with an analysis of the words contained in the title, abstract and indexed terms to describe the articles found. In a second stage, all the keywords and indexing terms in the chosen databases were used.

Studies published in Portuguese, English or Spanish were considered, with the application of limiters (abstract available, full text and time limit between 2018 and 2024 to ensure access to the most recent scientific evidence).

The various terms indexed in each database – CINAHL and MEDLINE – were considered, as well as the elements of the PCC format of the research question. It is important to note that, in an attempt to find additional sources, we proceeded, in a third stage, to identify bibliographic references in the selected articles depending on the title and abstract of the article [11]. Given the small number of articles in both platforms that met the inclusion criteria, searches carried out on Google Scholar were also considered.

### Study selection

Due to the large number of articles, the initial search was subject to filters, namely age (“all infants” and language “Portuguese/English/Spanish”). Of the 774 articles found in the CINAHL Complete and MEDLINE Complete databases, 3 duplicate articles were eliminated. Of the 771 articles, a total of 3 articles were obtained that met the inclusion criteria based on the verification of titles/abstracts, age of the child over one year, reference to the perception of parents about domestic accidents, as well as health professionals.

Due to the relevance of the information for the scoping review, articles were identified in secondary sources (Google Scholar). Of the 2 articles found that could be read in full, 1 was eliminated because it addressed the age group of children over one year old, leaving one eligible article. Four articles were ultimately identified as eligible in the search.

**Figure 1:**
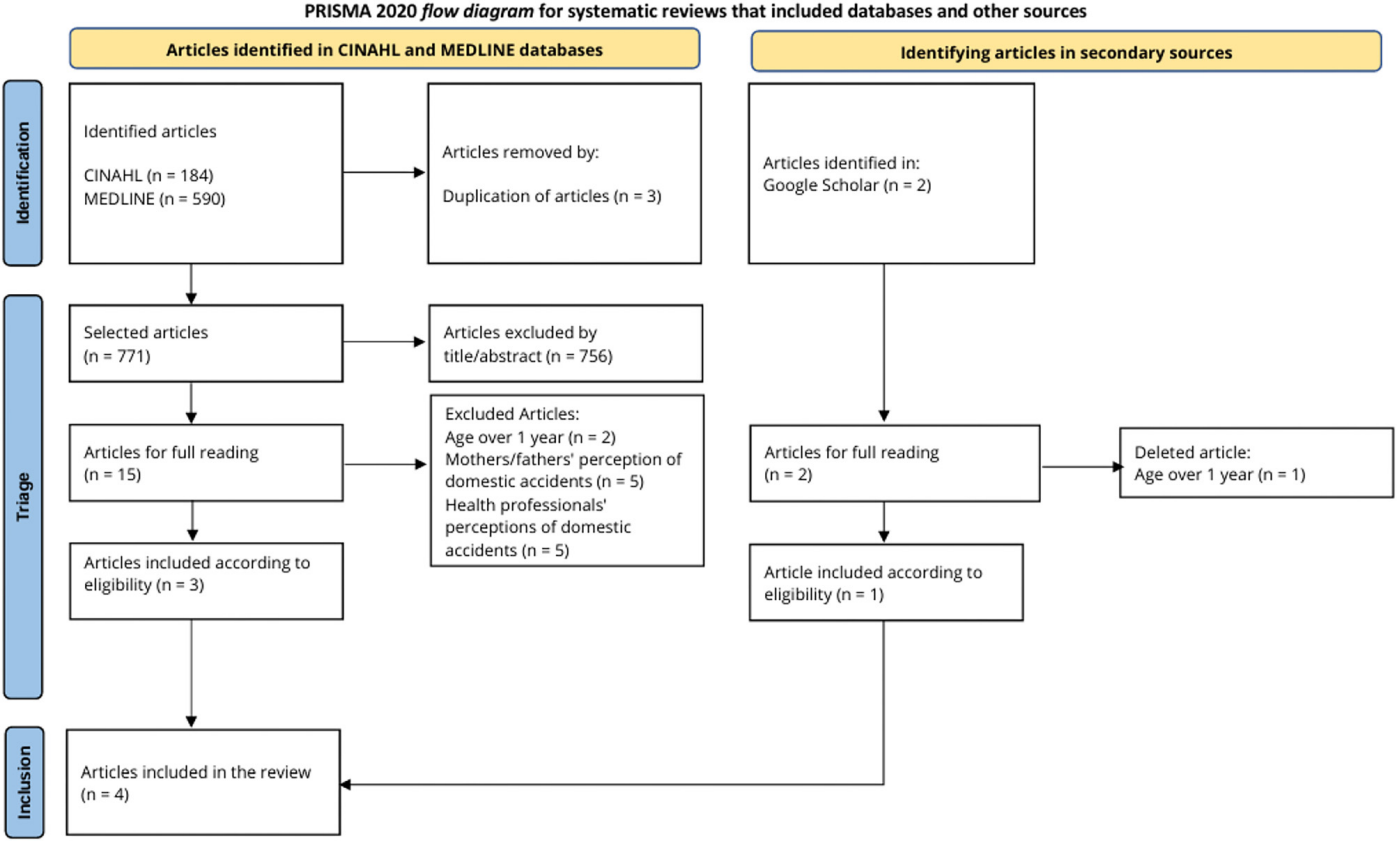
PRISMA flow chart.

Figure 1 summarizes the results of the analysis steps, following the PRISMA Flow Diagram [12].

Therefore, as already mentioned, 4 articles were selected for analysis, one of which was a quasi-experimental study (n=1), another a quantitative study (n=1) (both developed in Turkey) and the other two (n=2) are systematic reviews (South Korea and Iran).

Data extraction from the articles was carried out using an indexing table of results integrating the objective and the review question, which was organized by: study details (title, objective, research question, author(s), year of publication and country of study), inclusion/exclusion criteria (population, concept, inclusion criteria, exclusion criteria and types of sources of evidence) and results of the extraction of sources of evidence (characteristics of interventions and interventions for the prevention of domestic accidents) (Appendix I).

The extraction of the studies described in the articles was carried out by two reviewers independently, as was the synthesis of the data which is reflected upon below.

## Data analysis and discussion

The objective of this scoping review was to map the scientific literature on nursing interventions for preventing domestic accidents in families with children in their first year of life. To answer this question, four studies were included, two of which were primary studies. The importance of nurses in preventing accidents among children in the first years of life was found across the primary studies.

The interventions found referred to the role of the nurse within a family health team (quasi-experimental study) or the role of the nurse working with families (quantitative study).

In fact, the nurse assumes the role of collaborator by working in partnership with other health professionals to offer an integrated approach to the health situation of individuals and families, and also the role of leadership in projects, influencing health decisions and practices to improve family health and well-being [13]. In this approach, nursing performs autonomous and interdependent interventions. Autonomous actions are those carried out on the initiative and responsibility of nurses according to their respective professional qualifications. Interdependent actions are those carried out by nurses in conjunction with other professionals to achieve a common goal, resulting from action plans previously defined by multidisciplinary teams [14].

In the systematic reviews, there were no references to studies that mentioned the role of nurses, and the interventions were generalizable to any healthcare professional. However, one of them was published in a nursing journal, implying that accidents prevention was the focus of the nurse.

Considering that we found only two articles on the topic of nursing intervention, either as a member of the health team or in collaboration with the family, it affects the robustness of the study and reduces the generalizability of the findings, as it cannot specify for children aged 0–1 years, since the ages of the children in the study were between 0 and 6 years. This finding challenges the scientific community to investigate studies led by nurses with the aim of investigating the topic more specifically in the first year of life, given that the samples of the two eligible studies were in children under 6 years of age. The results, despite guiding the role of nurses in preventing domestic accidents among children, point to the scarcity of knowledge and scientific evidence on the subject and how investing in primary prevention will bring health gains for children, families, and globally for policymakers, health organizations, and society.

Regarding interventions related to the prevention of domestic accidents, there is a consensus on the importance of the place where they are carried out, that is, intervention in the domestic environment, also known as “home” [15], 16].

To understand the problem of domestic accidents, it is important to know and make known the types of accidents so that parents can take safety measures, particularly to prevent falls [16], 17]. One of the aspects related to the topic highlighted in the articles is knowledge of the characteristics of child development. It is therefore very important that nurses and parents are aware of these characteristics in order to adapt safety measures to the child’s stage of cognitive and motor development [16], 17].

Another variable to consider is family characteristics, which may influence risk factors such as the type of family (nuclear or extended); the number of children; the mother’s age (under 30 years); parental unemployment; and low level of literacy of parents [18].

There were authors who applied the “Risk Assessment Form for Pediatric Injuries” (RAF) to determine risk factors, as in the quasi-experimental study, the result being important to integrate into an intervention that aims to prevent or reduce this risk [16].

For most authors, domestic accidents can be prevented by changing the home environment, reducing the inherent dangers [16], 17].

Regarding the teaching methodology, emphasis is placed on strengthening the effectiveness of interventions focused on parents, giving as an example the organization of a box with safety products for demonstration in the domestic environment and programs that can be developed to improve parents’ competence in preventing domestic accidents [17]. Such programs allow for the feedback of information transmitted to parents, thus reinforcing the factors present in interventions to prevent domestic accidents.

Corroborating the previous information, other authors state that the teaching methodology could combine an educational approach with an active and demonstrative methodology with the use of safety equipment, for which there could be financial support for its acquisition [15].

Another intervention for preventing domestic accidents involves raising public awareness on this issue, using written and visual information through the media [16]. The publication of this type of information can include posters, leaflets (written), radio and television (visual) [18].

Interventions will be more effective if they are carried out systematically [17] and at specific time intervals [16]. Interventions every two weeks can consolidate the effect of previous interventions and improve their content and therefore their quality [17]. When these interventions are repeated daily over a period of 60 days, improvements in parental behaviour can be seen [17].

Finally, it is important to note that the success of any intervention depends on the voluntary participation of parents, with an appeal for participation by telephone or email [17]. Interventions should be organized according to the real needs and preferences of parents [17].

Corroborating what is recommended by authors [19] who state that individualized interventions are designed with and for the client and are characterized by: a) data collection that allows understanding the client’s preferences, needs and perceptions; b) individualized educational interventions and rehabilitation activities, taking into account the client’s physical and socio-environmental characteristics, their situation and reaction to their concerns; c) the client having control over the decision throughout the intervention, thus respecting individual expectations of having the power to participate in decision-making, to achieve the desired results.

## Conclusions

From the articles analysed focusing on nursing intervention, it was found that nursing interventions are relevant for the prevention of domestic accidents in children in the first year of life as a member of the health team in collaboration with the family, based on the effectiveness of parents in preparing their home environment, as well as in identifying and reducing risk factors for the occurrence of domestic accidents.

The motor and cognitive development phase is very rapid, especially in children in the first year of life. Therefore, it is urgent to train families to prevent domestic accidents, as a way to avoid serious situations for both the child and the family, with repercussions on achieving health gains.

The nursing interventions include a variety of actions supported by methodologies that should reinforce the parental role. These methodologies include the educational component, with the transmission of the type of domestic accidents, the risk factors for the occurrence of domestic accidents, knowledge of the child’s cognitive and motor development, preventive measures for each type of domestic accident and finally the first aid to be provided in the event of a domestic accident. These interventions should be systematic to strengthen both parental competence and previously transmitted interventions.

The research did not find articles written in Portugal related to this topic, however, the two primary studies from Turkey offer a variety of interventions transversal to the professional practice of nurses.

However, it is important to highlight the need to develop studies at a national and international level with samples of families with children up to one year of age, directing interventions to nurses based on the guidelines issued by national health policies as well as legislative guidelines.

There is a gap in knowledge on this topic. Indeed, despite its limitations, this scoping review aimed to contribute to the topic by presenting scientific evidence on fundamental aspects and guidelines for nurses in preventing accidents in families with children during the first year of life.

## Limitations

This scoping review has limitations due to the specificity of the topic and the relative scarcity of scientific evidence. One limitation is associated with two articles containing primary studies. Another was the language used for the search (Portuguese, English, and Spanish), as well as the time constraints that may have excluded potentially relevant articles. Another limitation was the search being limited to only two databases and the children’s age range being 0–1 year, which prevented generalizations and reduced the robustness of the study.

The limitations described above were considered necessary, both to reduce the number of articles and to allow for the most recent mapping of scientific evidence possible.

## Supplementary Material

Supplementary Material
