# Supplementary material for: Preventing domestic accidents in families with children: a scoping review
Source: Open Med (Wars). 2026 Jun 3;21(1):20261446. doi: 10.1515/med-2026-1446 (PMC13229063; doi:10.1515/med-2026-1446)
Supplement: Supplementary file 1 — Supplementary Material [file j_med-2026-1446_suppl_001.docx]

| **Study details** | **Inclusion/exclusion criteria** | **Results of extraction of evidence sources** |
| --- | --- | --- |
| **Title:** Analysis of research on interventions for the prevention of safety accidents involving infants: a scoping review  **Objective:** To conduct a scoping review of interventions for preventing safety accidents involving infants  **Research question:** What are the main findings of articles on preventing safety accidents involving infants and children in the home?  **Authors:** Kim M, Lee H, Lee Y, Kim J, Cho H.  **Publication year:** 2022  **Country of study:** South Korea | **Population:** children and parents  **Concept:** safety accidents and interventions  **Context:** home  **Inclusion criteria:**  journal studies related to interventions for the prevention of domestic accidents involving babies, studies published between 2012 and 2022, peer-reviewed studies, in English or Korean.  **Exclusion criteria:** editorials, letters to the editor, and conference proceedings  **Types of evidence sources:** scoping review | **Characteristics of interventions:**  Five characteristics identified: Precautions to prevent safety accidents at home; Characteristics of the child's developmental stages; Encouragement of voluntary participation (from parents); Continuity of interventions; Teaching methods that strengthen skills in preventing safety accidents.  **Interventions for preventing home accidents involving babies:**  Creating a safe home environment; Parental awareness of safety accidents in the home involving babies and children should be improved by the intervention; Interventions should ensure that parents are knowledgeable about safety accident prevention; Cognitive, physical and motor development; The messages to be transmitted can be sent by telephone or email, according to the needs and preferences of the parents; To ensure the success of the intervention, it must be repetitive and systematic and may lead to additional interventions; The continuity of the intervention strengthens the parents’ competence related to the prevention of safety accidents; Teaching methods should be used to strengthen the interventions, and a box containing safety products can be organized; Experience in managing safety at home; Development of programs that improve the parents’ competence regarding the prevention of safety accidents (feedback of practices). |

| **Study details** | **Inclusion/exclusion criteria** | **Results of extraction of evidence sources** |
| --- | --- | --- |
| **Title:** The Prevention of Non-Traumatic Home Accidents Among Children Aged 0-6 Year  **Objective:** Investigate the factors for domestic accidents and the prevention of these accidents in the 0-6 age group  **Research question:** What are the causes of domestic accidents in children aged 0-6 years and the factors that affect them and determine the methods of protection against domestic accidents?  **Authors:** Doğan M, Öztürk A.  **Publication year:** 2021  **Country of study:** Turkey | **Population:** children aged 0-6  **Concept:** domestic accidents  **Context:** home  **Inclusion criteria:** healthy children  **Exclusion criteria:** children with chronic diseases  **Types of evidence sources:** quantitative study | **Characteristics of interventions:**  Children assessed between January and December 2019, after admission to the pediatric emergency department due to a non-traumatic domestic accident. Application of a questionnaire to the family about the characteristics of the child (age, sex, etc.) and families (number of children, type of family, type of house, location of the accident, education, type of work), as well as the manner in which the accident occurred.  **Interventions for the prevention of domestic accidents in families with infants and children:**  Recommended education for mothers/families and caregivers to prevent domestic accidents regarding the developmental characteristics of children aged 0-6 years, risk factors for domestic accidents, safety measures to prevent domestic accidents and application of first aid.  Publishing educational material such as posters and leaflets and using the media (radio and television) on the prevention of domestic accidents. |

| **Study details** | **Inclusion/exclusion criteria** | **Results of extraction of evidence sources** |
| --- | --- | --- |
| **Title:** Effectiveness of interventions in the prevention of home injuries among children under 5 years of age: A systematic review  **Objective:** To summarize the literature on the effectiveness of interventions to prevent domestic accidents in children under 5 years of age.  **Research question:** not present  **Authors:** Abbassinia M, Barati M, Afshari M.  **Publication year:** 2019  **Country of study:** Iran | **Population:** Children under 5 years of age  **Concept:** Domestic accidents, prevention of unintentional injuries  **Context:** Home  **Inclusion criteria:** Randomized controlled trials to reduce and prevent domestic accidents in children under 5 years of age, including mothers, families and fathers of children, in English and Persian languages ​​and without limitation of year of publication.  **Exclusion criteria:** Descriptive, quantitative, reviews, systematic reviews, meta-analyses and quasi-experimental studies. Studies for the prevention of unintentional injuries in children with other interventions related to health behaviors, studies in preschool, studies with sick children or with some disability, studies on children under violence and abuse, interventions on psychological conditions in the prevention of injuries and studies on intentional injuries in children.  **Types of sources of evidence:** systematic review | **Characteristics of interventions:** Active interventions (practical sessions) or a combination of intervention measures are more effective in reducing the risk of domestic accidents and injuries in children than passive interventions.  **Interventions for preventing domestic accidents in families with infants and children:** Practical home sessions, home visits, group discussions and interviews as educational tools. Videos and pamphlets are also recommended as educational tools. A technological/engineering approach could be taken to provide safety equipment (with funding). |

| **Study details** | **Inclusion/exclusion criteria** | **Results of extraction of evidence sources** |
| --- | --- | --- |
| **Title:** Effects of a Safety-Awareness-Promoting Program Targeting Mothers of Children Aged 0-6 Years to Prevent Pediatric Injuries in the Home Environment: Implications for Nurses  **Objective:** Identify the effects of teaching provided to mothers with children aged 0-6 years about the dangers that lead to pediatric injuries  **Research question:** not present  **Authors:** Kahriman I, Karadeniz H.  **Publication year:** 2018  **Country of study:** Turkey | **Population:** mothers of children aged 0-6 years  **Concept:** perception and knowledge about prevention of pediatric injuries  **Context:** home environment  **Inclusion criteria:** mothers with children aged 0-6 years registered at the Family Health Centers of Beşirli, Fatih and Değirmendere  **Exclusion criteria:** children over 6 years old and mothers who are not registered at the Family Health Centers  **Types of evidence sources:** quasi-experimental study | **Characteristics of the interventions:** Three stages with the training of 60 senior nursing students. In the first stage, health experts provided training to the students on domestic accidents for a total of 16 hours. After the theoretical component, the practical component was carried out with simulations of domestic environments (living room, kitchen, bathroom and stairs) with situations that could lead to domestic accidents. The students who were able to identify the risk factors went on to the next stage. In the second stage, the students visited their mothers and the sociodemographic questionnaire, the RAF scale, was apply – “Risk Assessment Form for Pediatric Injuries” and “0-6-year-old Children´s Mothers´Identification Scale of Safety Precautions for the Prevention of Pediatric Injuries”. In the third stage, the students held a session focused on risk situations for domestic accidents and how to prevent them. During the session, posters and leaflets developed by the researchers were used to increase social awareness and improve the knowledge and behavior of the participating mothers. A month later, the scales were applied again.  **Interventions for the prevention of domestic accidents in families with babies and children:** information on the types of domestic accidents and preventive measures, characteristics of child development, changes in the domestic environment to reduce risks using educational material according to the theme. |
